# Prebiotics for depression: how does the gut microbiota play a role?

**DOI:** 10.3389/fnut.2023.1206468

**Published:** 2023-07-06

**Authors:** Yongde Yang, Bi Zhou, Sheng Zhang, Liang Si, Xiaobo Liu, Fu Li

**Affiliations:** Affiliated Wuhan Mental Health Center, Tongji Medical College, Huazhong University of Science and Technology, Wuhan, China

**Keywords:** depression, prebiotics, microbiota, mood disorder, Fiber

## Abstract

Depression, a mood disorder characterized by persistent feelings of sadness and aversion to activity that can interfere with daily life, is a condition of great concern. Prebiotics, which are non-digestible substances selectively utilized by host microorganisms for health benefits, have gained attention for their potential to improve overall wellness and alleviate various disorders including depression. This study aims to review clinical trials utilizing carbohydrate-type prebiotics such as inulin-type fructans, galactooligosaccharides (GOS), human milk oligosaccharides, resistant starch, prebiotic phytochemicals including epigallocatechin gallate (EGCG), chlorogenic acids, resveratrol, and prebiotic lipids (n-3 polysaturated fatty acids) to determine their effects on depression. Our findings suggest that GOS at a daily dosage of 5 g and eicosapentaenoic acid at or less than 1 g can effectively mitigate depressive symptoms. While EGCG exhibits potential antidepressant properties, a higher dosage of 3 g/d may be necessary to elicit significant effects. The plausible mechanisms underlying the impact of prebiotics on depression include the synthesis of neurotransmitters, production of short-chain fatty acids, and regulation of inflammation.

## 1. Introduction

**Figure 1:**
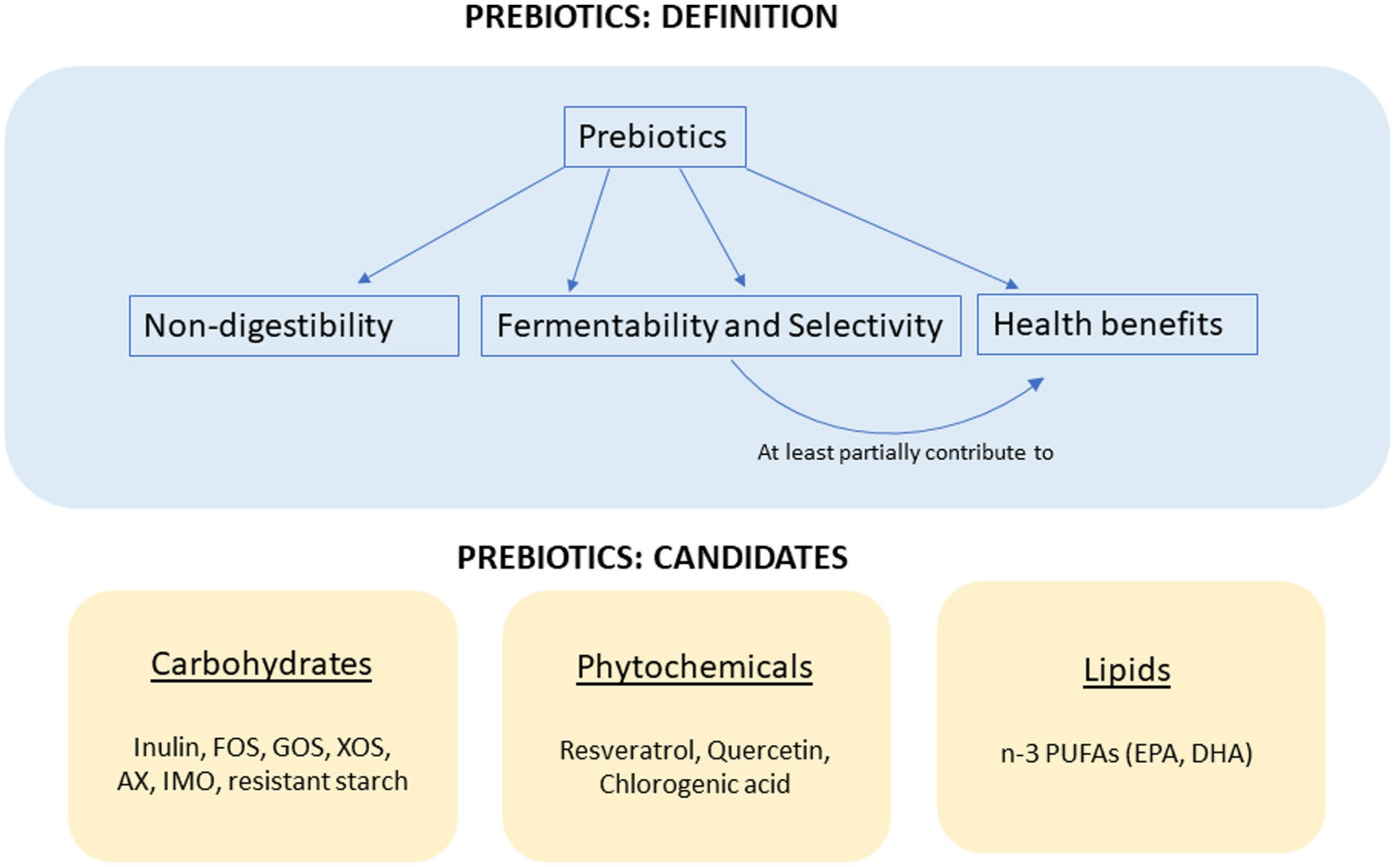
The definition of prebiotics and the candidates of prebiotics.

According to the International Scientific Association for Probiotics and Prebiotics, a prebiotic is defined as “a non-digestible substance that is selectively utilized by host microorganisms conferring a health benefit” (1). In addition, the health beneficial effects from the prebiotic need to be partially caused by microbial changes. To conclude, the four major elements for the consensus definition of prebiotics include nondigestibility, fermentability and selectivity, a health-related beneficial effect, and microbiota-mediated mechanism (Figure 1). Since the “prebiotic” concept was first put forward by Gibson and Roberfroid (2), the physiological benefits of prebiotics have been widely explored, especially in nutrient absorption, glucose management, lipid metabolism, and immune modulation.

Depression is one of the most common mental and mood health conditions, with an estimated 3.8% of the global population affected, generating negative consequences such as weight gain, inability to take care of oneself, impaired cognitions, and even suicidal ideations (3). In consistent with these findings, the lost productivity caused by depression and anxiety costs the global economy $1 trillion each year, and the economic burden of depression is projected to increase (4). In light of this, the World Health Organization released Mental Health Action Plan 2013–2030 to emphasize the importance of providing appropriate interventions for patients with mental disorders including depression.

The pathophysiology of depression remains unclear due to its etiological heterogeneity. In recent years, accumulating evidence have shown that depression is linked to dysbiosis by the gut-microbiota-brain axis (GBA) (5, 6), which is a bidirectional network that connects the gastrointestinal (GI) system and the central nervous system through the interplay of multiple neuroimmune and neuroendocrine pathways (5). Despite a lack of deep understanding in the causal relationship between microbial changes and the development of diseases, the last decade witnessed a growing interest in utilizing microbiome manipulation to alleviate depressive disorders. Of several approaches that have garnered considerable interest, prebiotics attracted substantial attention for their broad applications in food and beverages, potential functionalities in improving texture (7), being free of addictive properties and generating less adverse effects (8).

There have been several review articles that discussed the role of prebiotics in depression (9–12). However, some of these publications lack mechanistic discussions on how prebiotics may alleviate depression, or mainly focused on dietary fibers and mental health instead of prebiotics and depression. Additionally, all these publications focused on carbohydrate-type prebiotics, whereas non-carbohydrate food ingredients that are also qualified for a prebiotic claim, such as fermentable phytochemicals (13), were left out from the studies. Chudzik et al. (11) synthesized the empirical evidence of pre-, pro-, and post-biotics for depressive symptoms, which provided the latest review on this topic. Nevertheless, with multiple animal studies and human studies published in the year 2022, it would be valuable to revisit the literature and update our knowledge.

Considering these, the objective of the current work is to summarize the animal and human studies that investigated the impact of prebiotics intake on depressive disorders. Since microbiota-mediated mechanism is a requisite for defining a substance to be prebiotic, this project highlights how the gut-brain-axis (GBA) moderates the effects of prebiotics in depression.

## 2. Prebiotics and their impact on the gut microbiota

The human gut harbors 10–100 trillion microorganisms, forming a dynamic and complex ecosystem (14). Within a “reference man” that weighs 70 kg, the total number of bacteria is approximately 3.8 × 10^13^, and the ratio between human cells and resident microbes is approximately one-to-one (15). The gut microbiota is taxonomically classified by phyla, order, family, genus and species. Their genome consists of approximately over 3 million genes, encoding a large repertoire of biochemicals as a way of affecting the hosts’ health and susceptibility to diseases (16). The gut microbiota can be altered by various environmental cues such as medication, health status, physical activity, as well as diets (16). Prebiotics hold substantial appeal due to their capability of selective fermentation, meaning that these compounds will exclusively stimulate the growth of beneficial bacteria, not pathogenic bacteria. Naturally occurring prebiotics and their respective food sources were listed in Table 1.

**Table 1:** Prebiotics and food sources.

Prebiotics	Food sources	Microbiota stimulated
**Carbohydrates**		
Inulin	Onion, banana, garlic, leek, wheat, asparagus, Jerusalem artichoke, chicory, and the blue agave plant (11, 17, 18)	*Bifidobacterium*, *Anaerostipes, Faecalibacterium, and Lactobacillus*
FOS		*Bifidobacterium*
GOS	Legumes, *Lycopus lucidus* and certain Traditional Chinese Medicine herbs (19–21)	*Bifidobacterium*
XOS	Fruits and vegetables, bamboo shoots, milk, and honey (22)	*Bifidobacterium*
AX	Major cereal grains (23)	*Bifidobacterium*
IMO	Miso, sake, soy sauce, and honey (22)	*Bifidobacterium*
RS	Starchy fruits and vegetables, legumes, cereal grains, and seeds (24), or synthesized starch	*Ruminococcus* and *Parabacteroidetes*
**Phytochemicals**		
Resveratrol	Grapes, wine, grape juice, peanuts, cocoa, and berries of Vaccinium species, including blueberries, bilberries, and cranberries (25–28)	*Bifidobacterium*
Quercetin	Onions, kale, apples, cherries, and red wine (29)	*Bifidobacterium*
Chlorogenic acid	Apples, artichoke, betel, burdock, carrots, coffee beans, eggplants, eucommia, grapes, honeysuckle (30)	*Bifidobacterium*
EGCG	Green tea	*Akkermansia*, *Bifidobacterium*
**Lipids**		
n-3 PUFAs	Marine organisms or deep-sea fish (31)	*Lachnospiraceae*, *Bacteroidetes*, *Roseburia*, *Coprococcus*, and *Blautia*

Inulin and inulin-type fructans, such as fructooligosaccharides (FOS), consist repetitive β–(2,1) fructosyl-fructose glycosidic linkages that are undigestible by the human intestinal brush boarder enzymes but readily fermentable by the gut microbiota (17). Although the chain length of inulin, FOS, and short-chain FOS differ, these compounds exhibit similar biological effects due to the same type of glycosidic linkage. The most consistently reported microbial alteration with the consumption of inulin and FOS is the increase of *Bifidobacterium* (32, 33), and other concordant data indicate that inulin supplementation may stimulate the growth of *Lactobacillus*, *Faecalibacterium*, and *Anaerostipes* (33). Galactooligosaccharides (GOS), xylooligosaccharides (XOS), arabinoxylan (AX) and Isomaltooligosaccharides (IMO) are considered prebiotics with their bifidogenic effects (22, 34–36). All of the above-mentioned oligosaccharides are naturally contained in food, but they can also be prepared by using enzymatic or chemical methods (17, 22, 37). While other soluble fibers such as β-glucan and konjac glucomannan oligosaccharides have displayed certain prebiotic features, there is insufficient evidence that linked their physiological benefits to microbial changes. Resistant starch (RS) refers to a group of non-soluble carbohydrates that are resistant to upper GI digestion. RS is divided into four categories: RS1 is physically protected from digestive enzymes; RS2 is native granular starch consisting of a high amount of amylose; RS3 is retrograde starch; and RS4 is chemically modified starch. Notably, although RS are generally insoluble, certain RS molecules can be fermented by the gut microbiota, particularly by *Ruminococcus bromii* and *Parabacteroides distasonis* (38).

In addition to carbohydrates, certain phytochemicals are considered as prebiotics (13), since they are able to confer health benefits by selectively promoting the healthy gut microbiota. Resveratrol, quercetin, and chlorogenic acid, exemplified the most-studied phytochemicals that have shown health-promoting effects by increasing the abundance or proportional representation of *Bifidobacterium* strains and, therefore, are recognized as prebiotics (39). Besides, quercetin may reduce the opportunistic or pathogenic bacteria including *Listeria monocytogenes*, *taphylococcus aureus*, and *Vibrio parahaemolyticus*, which are clinically significant bacteria that may cause infection or diseases (29). Epigallocatechin gallate (EGCG), another well-studied phytonutrient, was found to enrich *Bifidobacterium* (40) and the SCFA-producing microbiota, such as *Akkermansia*, and exerted potent anti-inflammatory and anti-oxidative effects through enhancing the gut SCFA concentrations (41).

Intestine is the major site where lipid digestion and absorption occur. Lipid absorption is a complex process, which is almost fully completed in the small intestine. The lipids appearing in the colon and fecal materials are partly from dietary lipids that escape digestion in the upper GI system. As estimated, 95% consumed lipids are absorbed in the jejunum and ileum, leaving only approximately 5% dietary lipids entering the large intestine for further bacterial fermentation. Even with such a small quantity, the certain undigested lipids are able to induce changes in the gut microbiota. Omega-3 polyunsaturated fatty acids (n-3 PUFAs) are essential fatty acids because the limited quantity of *de novo* n-3 PUFAs synthesis is insufficient to satisfy the needs of humans. Eicosapentaenoic acid (EPA) and docosahexaenoic acid (DHA), are long-chain n-3 PUFAs that are abundant in marine organisms or deep-sea fish, such as salmon, mackerel, and sardines (31). The influence of n-3 PUFAs on the gut microbiota was not widely studied. However, in the limited studies on adults, the authors showed a consistent effects of n-3 PUFAs in modulating the gut microbiota, with a decrease in *Faecalibacterium* and increase in *Lachnospiraceae* (42), *Bacteroidetes*, *Roseburia*, *Coprococcus*, and *Blautia*, which were associated with an enhanced production of SCFAs (43). Both DHA and EPA may attenuate multiple diseases by directly or indirectly affecting the gut microbiota (31). Conjugated linoleic acids (CLA) are isomers of linolenic acid and are heavily enriched in foods such as meat and dairy products (44). Multiple animal studies reported that CLA could modulate the gut microbiota, especially in promoting *Prevotella, Akkermansia muciniphila* (45), *Lachnoclostridium*, *Roseburia*, *Dubosiella*, *Oscillibacter*, and *Anaerostipes* (46), as well as harboring a higher proportion of *Bacteroidetes* phylum in general (47). However, the efficacy of CLA on the gut microbiota has not been investigated in any clinical trials. The taxonomy of the promoted bacteria varied based on different animal models and different CLA isomers, causing a huge homogeneity in evidence. Therefore, more studies, especially in humans, are warranted to explore the efficacy of CLA in modulating the gut microbiota. Interestingly, certain gut microbiota, such as *Lactobacillus*, *Butyrivibrio*, and *Megasphaera* can feed on undigested linoleic acids and produce CLA (48, 49), which demonstrates a bidirectional relationship between CLA and the gut microbiota.

## 3. Depression and dysbiosis

Dysbiosis describes an imbalanced microbial profile characterized by loss of beneficial microbial abundance or signal and an augmentation of opportunistic and pathogenic microflora. Although the study of gut microbiota and mental health is a relatively new area that has caught researchers’ attention in the past few years, converging clinical data already showed that patients diagnosed with depression may experience gut microbiome dysbiosis (50). The correlation between dysbiosis and depression has been portrayed in several observational studies. An overrepresentation of *Bacteroidales*, and an underrepresentation of *Lachnospiraceae* families, within the phylum *Firmicutes*, were observed in depressive patients (51). Patients with major depressive disorder (MDD) showed increased fecal bacterial α-diversity and enhanced *Bacteroidetes*, *Proteobacteria*, and *Actinobacteria*, but decreased *Firmicutes*. In particular, these patients had increased levels of *Enterobacteriaceae* and *Alistipes*, whereas their levels of *Faecalibacterium* were reduced and were negatively correlated with depressive symptoms (52). These results were, to a certain degree, consistent with the study by Naseribafrouei et al. (51) showing enrichment of the *Alistipes* in the subjects with depressive symptoms, indicating that depression patients may have a higher risk of undergoing dysbiosis.

Vice versa, the incidence of depression and other mental conditions were significantly higher in the patients with inflammatory bowel disease (IBD) than the subjects who had a healthy GI system (53, 54). A study by Chung et al. (55) explored the association between dysbiosis and depression by retrospectively utilizing a cohort where adult patients who were diagnosed with dysbiosis (*N* = 552) and their healthy counterparts (*N* = 52) were followed up for 5 years. The researchers found that the incidence of depression within 5 years of the index data was significantly higher in the patients who were diagnosed with dysbiosis, compared with the healthy controls (HR = 2.85) (55). Interestingly, the association was more potent in males than in females. Age was another important variable that modulated the degree of association, with a stronger association observed in the age group more than 60 years old (55). However, this retrospective study, along with the other observational studies, did not show causal relationships between the gut microbial changes and the onset or development of depression; instead, they focused solely on associations.

## 4. Prebiotics for depression

Since dysbiosis was closely linked to depression, and prebiotics are functional to alter the gut microbiota and mitigate dysbiosis, several well-designed clinical trials evaluated the effects of different prebiotics in alleviating depression. Clinical trials are the gold standard for evaluating the effectiveness of the compounds in certain conditions or diseases, as they represent the most rigorous method of examining causal relationships between interventions and outcomes.

### 4.1. Carbohydrates

Eleven clinical trials investigated the role of prebiotic carbohydrates in depression and depression-related parameters (Table 2). Among these studies, seven studies investigated inulin-type fructans that include inulin and FOS with different chain lengths. Three studies provided GOS treatment. The roles of HMO and RS in depression were investigated in one clinical trial, respectively.

**Table 2:** Prebiotic fibers for depression: study characteristics.

Study	*N* ^a^	Mean age (years)	Healthy status	Prebiotic	Dosage	Study duration	Clinical measures	Mood health parameter changes	Bacterial change
Smith et al. (56)	142	32.0	Healthy	Inulin-type fructan	10 g/d	2 weeks	HADS-A, HADS-D	Mood (positive, negative, or depression): NC; anxiety: ↑ Psychomotor performance: NC Sleep quality: NC Memory: NC	Not available
Silk et al. (57)	44	54.0	IBS patients	GOS	3.5 or 7.0 g/d	4 weeks	HADS-A, HADS-D	Anxiety: lower dosage NC, higher dosage ↓ Depression: lower dosage NC, higher dosage NC	Not available
Smith et al. (58)	47	23.0	Healthy	Inulin-type fructan	5 g (one-time)	4 h	SSM	Subjective feeling of happiness: ↑ Subjective feeling of indigestion: ↓ Mood rating: NC Memory tasks (immediate free recal, delayed recall): ↑; delayed recognition memory: better accuracy but slower reaction times. Logistic reasoning, semantic processing and spatial memory: NC	Not available
Schmidt et al. (59)	45	23.7	Healthy	GOS, inulin-type fructan	5.5 g/d	3 weeks	STAI	Cortisol awakening response: GOS↓, FOS NC Attentional vigilance to negative vs. positive information: GOS↓, FOS NC Emotional categorization, recall and recognition: GOS NC, FOS NC Self-report anxiety: GOS NC, FOS NC Self-report perceived stress: GOS NC, FOS NC	Not available.
Azpiroz et al. (60)	79	41.7	IBS patients	Inulin-type fructan	5 g/d	4 weeks	HADS-A, HADS-D	Anxiety: ↓ Depression: NC	Total anaerobes: NC ↑Bifidobacteria
Kazemi et al. (61)	72	36.7	Depressed patients	GOS	5 g/d	8 weeks	BDI-II	Depression: NC Biomarkers: kynurenine NC; tryptophan (Trp) NC; kynurenine/Trp ratio NC; Trp/BCAA ↑; Trp/isoleucine NC	Not available
Iribarren et al. (62)	60		IBS patients	HMO	5 or 10 g/d	4 weeks	HADS	Anxiety: NC Depression: NC	↑Bifidobacteria
Moludi et al. (63)	48	52.0	CAD patients	Inulin-type fructan	15 g/d	2 months	STAI BDI-II	Anxiety: NC Depression: NC	Not available
Vaghef-Mehrabani et al. (64)	45	39.8	MDD patients	Inulin-type fructan	10 g/d	8 weeks	STAI, STAII, HRDS, BDI-II	Anxiety: NC Depression NC	Not available
Amadieu et al. (65)	50	48.2	AUD patients	Inulin-type fructan	4–16 g/d	19 days	STAI, BDI	Anxiety: ↓ Depression: ↓	↓diversity and evenness Total anaerobes: NC ↑Actinobacteriota phylum, Bifidobacteriaceae family, Bifidobacterium genus; ↓Bacteroidaceae family, Bacteroides, Dorea and *Ruminococcus torques*
Becker et al. (66)	49	65.2	PD patients	RS	10 g/d	8 weeks	BDI-II	Depression: ↓	↑genus Rhodococcus

a Number of subjects included in the relevant statistical analyses.

AUD, alcohol use disorder; BDI, Beck depression inventory; CAD, coronary heart disease; IBS, irritable bowel syndrome; NC, not changed; PD, Parkinson’s disease; STAI, State-trait Anxiety Inventory; HADS, Hospital Anxiety and Depression Scale; HDRS, Hamilton Depression Rating Scale.

In a cross-over study, Smith et al. (56) recruited 153 participants, and randomized them into two groups: the interventional group was provided with 10 g/d inulin, whereas the control group was given a placebo powder for 2 weeks, followed by a 2-week washout. As a result, the inulin supplementation did not alter any mental health related biomarkers, including mood, sleep quality, and memory, except for the surprisingly increased anxiety score (56). In 2015, the same group of researchers performed another clinical trial, in which 47 subjects were included and provided with one-time, 5 g inulin. In this trial, the researchers reported that inulin supplementation significantly enhanced the subjective mood and cognitive performance, with the most substantial effects on the episodic memory tasks, including improved accuracy on a recognition memory task and better recall performance (58). Similar beneficial effects of inulin-type fructan in improving mood health were observed in another clinical trial that used 5 g/d short-chain FOS (scFOS) for 4 weeks among 79 IBS patients with rectal hypersensitivity. In this study, researchers found that scFOS supplementation significantly decreased anxiety but did not change depression. Intriguingly, the subjects who took scFOS also experienced less rectal sensitivity and increased *Bifidobacteria*, indicating that scFOS might reduce anxiety by improving GI health (60). Among 48 patients with coronary heart disease, inulin supplementation at 15 g per day for 2 months did not affect patients’ anxiety or depression scores, but the authors reported that the addition of inulin to a probiotic consisting of 1.9 × 10^9^ colony-forming unit (CFU) of *L. rhamnosus* significantly improved psychological outcomes including decreased BDI and anxiety state (63). In a recent publication, researchers investigated the role of inulin on MDD patients at a dosage of 10 g/d for 8 weeks but failed to observe any significant effects in depression, but a trend of decreased anxiety score by using State-trait anxiety inventory II (STAII) (64). A pilot study which included 50 patients with alcohol use disorder depicted significant anti-depression and anti-anxiety effects of inulin supplementation for 19 days. The dosage of inulin was increased gradually from 4 to 16 g per day to reduce the GI adverse effects (65). Another clinical trial included 45 subjects and randomized them to receive one of two prebiotics (FOS, *N* = 15; Bimuno GOS, *N* = 15), or placebo at a dose of 5.5 g per day for 3 weeks. Results showed that GOS significantly decreased cortisol awakening response and attentional vigilance to negative vs. positive information, suggesting that GOS at 5.5 g per day may have anxiolytic effects by suppressing the neuroendocrine stress response. The administration of FOS did not generate any significant effects (59). In a crossover study where 44 IBS patients were included, the researchers found that GOS supplementation at both 3.5 and 7.0 g per day significantly alleviated IBS symptoms, but only the higher dosage significantly improved the anxiety scores without changing the depression severity (57). Consistently, a study with 72 depressed patients failed to observe significant anti-depressive effects of GOS at 5 g per day for 8 weeks, despite that GOS supplementation significantly increased the tryptophan to BCAAs ratio (61). These pieces of evidence led us to conclude that both inulin-type fructans and GOS have limited effects in depression on both healthy and diseased population, even though FOS appeared to exhibit certain acute anti-depressive benefits. However, it is intriguing that GOS supplementation consistently decreased the level of anxiety in different studies, which warrants to be further discussed.

The implications of HMO in psychological modification were investigated in one study with 60 IBS patients. At 10 g per day, 4-week HMO treatment significantly enhanced the abundance of Bifidobacteria, but did not affect anxiety or depression scores (62).

In an open-label clinical trial, researchers investigated the effects of an eight-week treatment with RS (type 3) at 10 g per day, and found the treatment to be effective in reducing depression scores and enhancing fecal butyrate levels (66). Nevertheless, this is the first and single study that we identified in exploring RS and depression. Therefore, translation requires caution as the totality of evidence is not sufficient to conclude the efficacy of RS in modulating mood health.

### 4.2. Phytonutrients

The benefits that phytonutrients can contribute to human health have been explored by various studies, targeting on a wide range of endpoints including cognitive performance and mood. In total, four studies focused on resveratrol, two studies used chlorogenic acid, and two studies leveraged EGCG treatments (Table 3).

**Table 3:** Prebiotic phytonutrients for depression: study characteristics.

Study	*N* ^a^	Mean age (years)	Healthy status	Prebiotic and dosage	Study duration	Clinical measures	Mood health parameter changes
Scholey et al. (67)	31	27.7	Healthy	EGCG: 300 mg	180 min	MVAS	Calmness ↑, stress ↓ Alertness, contentedness, fatigue: NC
Wightman et al. (68)	27	22.0	Healthy	EGCG: 135 or 270 mg	90 min	MVAS	Relaxation, alertness, jitters, tiredness, tense, mental fatigue: NC
Camfield et al. (69)	40	64.5^b^	Healthy	Chlorogenic acids: 540 mg	120 min	MVAS	Jitters and headache: ↓ Tiredness, alertness, clam, relaxation, mental fatigue, overall mood score: NC
Witte et al. (70)	46	64.3	Overweight	Resveratrol: 200 mg/d	26 weeks	BDI	Depression: ↓
Wightman et al. (71)	23	21.0	Healthy	Resveratrol: 250 mg/d	One-time	MVAS	Alert, jitters, mental fatigue, relaxed, tense, tired: NC Overall mood: NC
Evans et al. (72)	80	61.5	Healthy	Resveratrol: 150 mg/d	14 weeks	CES-D	Anxiety: ↓ Depression, anger, fatigue, confusion, vigor: NC
Köbe et al. (73)	40	67.2	MCI patients	Resveratrol: 200 mg/d and quercetin: 350 mg/d	26 weeks	BDI STAI-X1	Anxiety: NC Depression: NC
Enokuchi et al. (74)	82	51.0	Menopausal symptoms	Chlorogenic acids: 270 mg/d	4 weeks	STAI	Anxiety: NC

a Number of subjects included in the relevant statistical analyses.

b Median age.

BDI, Beck’s Depression Index; MCI, mild cognitive impairment; NC, not changed; MVAS, mood visual analog scales; STAI, State-trait anxiety inventory.

Among 40 patients diagnosed with mild cognitive impairment, a mixture of resveratrol (200 mg/d) and quercetin (350 mg per day) for 26 weeks did not induce change in anxiety or depression (73). The independent effects of resveratrol in mood health were reported by three clinical trials. Evans et al. (72) recruited 80 postmenopausal women and randomized them to take *trans*-resveratrol (150 mg per day) or placebo for 14 weeks. The researchers found resveratrol supplements to be effective in reducing anxiety, but not functional in modulating depression, anger, fatigue, confusion, or vigor (72). The acute efficacy of resveratrol in modulating mood also appeared to be minor, as a group of researchers found that 250 mg resveratrol did not induce any changes in the subjective feelings of being alert, jittery, fatigue, relaxed, tense, or tired (71). The only clinical trial that reported a positive effect of resveratrol in depression was the one by Witt et al. (70), in which the 23 overweight subjects were provided resveratrol supplementation at 200 mg per day for 26 weeks and as a result, the supplementation was effective in reducing depression scores. However, the control group of this study was pairwise-matched to the intervention group, meaning that randomization and double-blinding were not achieved. Therefore, by summing up the above-mentioned clinical trials, the efficacy of resveratrol in modulating mood health including depression is weak and inconclusive.

Although the antidepressant effects of quercetin have been investigated in multiple animal studies, currently, few clinical trials have leveraged quercetin as a nutritional approach to improve depression. Interestingly, used as a part of the ingredients in the traditional Chinese medicinal formula, quercetin was reported to be functional in relieving depression by several clinical trials, and its efficacy even appeared to more potent than traditional antidepressants such as fluoxetine, paroxetine, and duloxetine (75).

Camfield et al. (69) examined the effects of a decaffeinated pure chlorogenic acids on the cognitive and mood performance of healthy adults aged 50 years or older. The participants were given either the chlorogenic acids or placebo in a double-blind acute cross-over design, and their cognitive and mood responses were assessed at pre-dose, 40-min and 120-min post-dose. While the study found no significant effects on the primary cognitive outcome measure, it did observe some improvements in jittery and headache with the chlorogenic acids in comparison to the placebo (69). In a study among healthy women with menopausal symptoms, researchers assigned the subjects to take either placebo or chlorogenic acids at a daily dosage of 270 mg for 4 weeks. Although findings showed significant improvements compared to the baseline values in both groups, no significant difference between the two groups was observed.

The role of EGCG in mood health was examined by two clinical trials. In the study that was conducted by Wightman et al. (68), 27 healthy adults received a placebo and two different doses (135 and 270 mg) of EGCG on separate days in a counterbalanced order. Their results showed that EGCG did not have significant effects in modulating relaxation, alertness, jitters, tiredness, tense and mental fatigue (68). However, in another study, Scholey et al. (67) found EGCG supplementation at 300 mg was effective in improving calmness and decreasing stress but did not affect alertness, contentedness and fatigue. Although insufficient data were available to substantiate EGCG’s psychopharmacological effects, preliminary evidence suggests that EGCG may have a calming effect during the second hour post-dose. Given the fact that Scholey et al. (67) utilized a higher dosage in the study, it is possible that EGCG needs a higher dosage to exert its beneficial effect. However, such hypothesis needs to be further investigated in a dose-respondent study.

### 4.3. n-3 PUFAs

There is an increasing amount of evidence suggesting that omega-3 polyunsaturated fatty acids (omega-3 PUFAs) are effective in improving depression. The summary of the studies was based on a publication (76) that systematically summarized 26 studies, which included 2,160 subjects. The results showed that EPA and a formula with ≥60% EPA were effective in decreasing depression, and it was found that an EPA dosage ≤1 g/d resulted in significant benefits. On the contrary, DHA and a formula with ≥60% DHA did not show significant effects in alleviating depression. In addition to the effects in treating depression, PUFAs showed preventive effects against depression. A systematic review and meta-analysis of 20 RCTs on 7,682 participants showed that n-3 PUFAs supplementation showed a modest beneficial effect on depressive symptomology compared to the placebo groups. In spite of a potential publication bias, the subgroup analysis of this study indicated that longer treatment duration may improve the effectiveness of n-3 PUFAs in preventing depression, especially depression with a lower degree of severity. However, a difference in favor of lower EPA dosage was observed, and the quality of evidence is relatively low (77). Several studies were published after the publication of these two meta-analyses. In a clinical trial with 61 MDD with high inflammation, only the 2 g/d dose of EPA decreased peripheral blood mononuclear cell tumor necrosis factor alpha (TNFα) levels, and EPA 4 g/d had a medium effect size for response rates compared to placebo. The study suggests that EPA 4 g/d may be effective in alleviating MDD in overweight individuals with elevated inflammatory markers, and changes in hs-CRP may be correlated with clinical response (78). The effects of a high n-3 plus low n-6 (H3-L6) dietary intervention in mood health was examined in a study with bipolar disorder (BD) patients, and the control diet contained usual U.S. level of n-6 and n-4 PUFA intakes. The results showed that H3-L6 group had reduced variability in mood symptoms compared to the control group, suggesting that the dietary intervention may be effective in improving mood stability in BD (79). One study aimed to investigate the effects of fish oil, which contains omega-3 polyunsaturated fatty acids, on emotion-generated corticolimbic functional connectivity in depressed youth at high risk for bipolar I disorder. The results showed that fish oil increased erythrocyte EPA + DHA composition and altered functional connectivity between the orbitofrontal cortex and superior temporal gyrus, and between the amygdala and inferior temporal gyrus. These changes were correlated with decreases in the severity of bipolar I disorder (80).

Apolipoprotein E (ApoE) is a protein involved in the metabolism and transport of lipids in the body. It is primarily produced in the liver, but it is also synthesized by other cells, including macrophages and neurons in the brain. ApoE plays a crucial role in the transportation of cholesterol and other lipids through the bloodstream and the regulation of lipid homeostasis (81). In elderly, a combination of 1,491 mg DHA and 351 mg EPA per day was effective in modulating the function of APOE ɛ4 carriers on depression and anxiety scores (82). APOE ɛ4 is a variant of the APOE gene that is associated with an increased risk for developing Alzheimer’s disease. In terms of mood and cognition, APOE ɛ4 carriers have been found to have a higher risk for developing depression and anxiety compared to non-carriers (83). People with APOE ɛ4 may also experience cognitive decline at a younger age compared to non-carriers, although not all APOE ɛ4 carriers will develop Alzheimer’s disease or experience significant cognitive decline (84). It is important to note that while APOE ɛ4 may increase the risk for certain health outcomes, it does not determine them. Environmental factors, lifestyle choices, and other genetic factors can also play a role in determining an individual’s overall health and cognitive function (85).

## 5. Mechanism: gut-brain-axis moderates the effects of prebiotics in depression

The gut microbiota, referred to as the “second brain” in humans due to its regulatory impact on the central nervous system through neuronal, chemical, and immune pathways, has been shown to establish a bidirectional communication channel between the gut and the brain (86). The gut-brain-axis (GBA) was discovered when gastrointestinal endocrine system changes were linked to alterations in neurons and brain cells. This concept was supported by studies that showed the contribution of gut microbiota to the development of cognitive diseases. Over 20 years ago, the observation of significant improvement in patients with hepatic encephalopathy after receiving oral antibiotics provided the most convincing evidence of a connection between gastrointestinal microbes and the brain in humans (87). Recently, emerging data suggested that the microbiota plays a role in influencing anxiety, depression, and other mood disorders. Patients with depression have different gut microbial profiles compared to healthy controls, while patients with gut inflammation experience mood and cognitive disturbances. *In vivo* studies show that gastrointestinal inflammation can induce anxiety-like behavior and alter the central nervous system (88). Germ-free animals have been used to determine causality, reporting that the absence of gut microbiota promotes anxiety and neuroendocrine response to stress (89). Prospective studies indicate the impact of dysbiosis on mental health disorders. Fecal microbiota transplantation studies have also shown that depressive symptoms can be transmitted to recipients, with those receiving fecal microbiota from depressed patients exhibiting more severe symptoms compared to those who received fecal microbiota from healthy subjects (90). These findings indicate a causal role of gut microbiota in the development of depressive symptoms.

### 5.1. Neurotransmitters

A growing body of evidence supports the notion that the interplay between the gut and brain, mediated by the gut microbiota, has been associated with the cause and development of depression and anxiety. Strains of Bifidobacteria, such as *B. adolescentis*, have the capability to produce Gamma-Aminobutyric Acid (GABA), a primary inhibitory neurotransmitter in the brain (91). In particular, *B. adolescentis* PRL2019 and *B. adolescentis* HD17T2H are the most distinguish GABA producers among the *B. adolescentis* strains (91). An animal study revealed that pretreatment with *B. adolescentis* resulted in anxiolytic and antidepressant effects in mice under chronic restraint stress. This was observed through an increase in time spent in open spaces, a decrease in immobility duration, and a reduction in inflammatory cytokine expression in the hippocampus, suggesting that the anxiolytic and antidepressant effects of *B. adolescentis* are related to its ability to reduce inflammation and rebalance the gut microbiota (92). In a study with children, researchers portrayed a negative correlation between *B adolescentis* and mental disorders, including depression and anxiety (93). It warrants further investigation whether the bifidogenic prebiotics function through enhancing GABA production.

**Figure 2:**
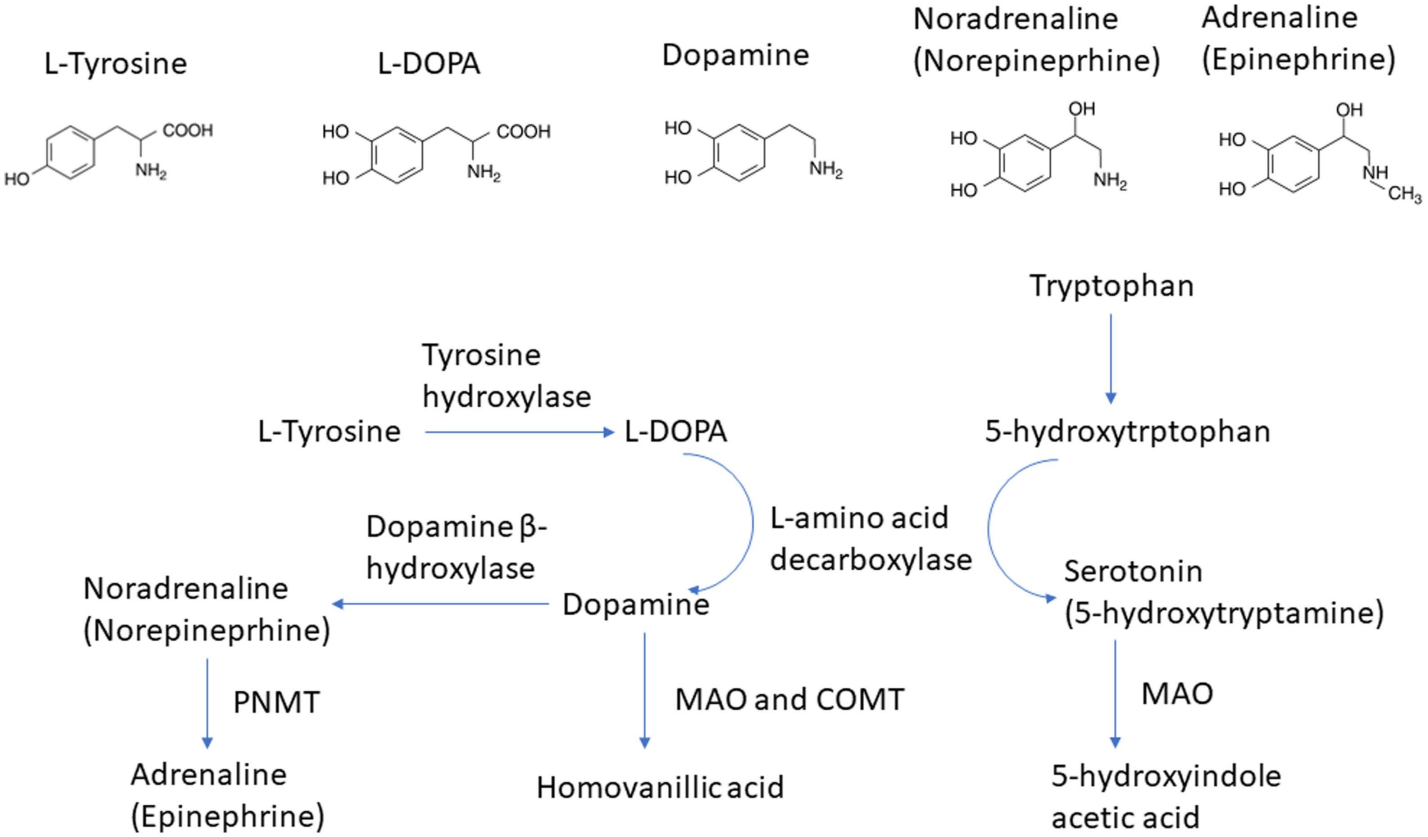
Pathway of norepinephrine and epinephrine biosynthesis.

In addition to GABA, other neurotransmitters including serotonin, norepinephrine, and dopamine can also be synthesized by the gut microbiota. Studies have demonstrated that *Streptococcus*, *Enterococcus*, and *Escherichia* are involved in the synthesis of the above-mentioned neurotransmitters (94–96). Norepinephrine, or noradrenaline, functions within the central nervous system and concurrently serves as a stress hormone. It contributes to the “fight or flight” response and is linked to mechanisms of arousal, attention, and focus (97). Dopamine partakes in reward processing and motivation, alongside movement control. It is affiliated with sensations of pleasure and contributes to the progression of addiction (98). The capacity of dopamine to act as a precursor in the biosynthesis of norepinephrine and epinephrine is well established in scientific literature (Figure 2). Serotonin, also known as 5-hydroxytryptamine (5-HT), is involved in regulating mood, appetite, sleep, and other bodily functions (97). Based on preclinical and clinical findings, it has been established that the dopamine, 5-HT, and norepinephrine in the central nervous system are disrupted in individuals experiencing depression (97, 99). Presently available antidepressants function mainly through one or more of the following mechanisms: hindering the reuptake of serotonin or norepinephrine, obstructing inhibitory presynaptic serotonin or norepinephrine receptors, or inhibiting monoamine oxidase (99). All of these mechanisms lead to elevated concentrations of serotonin and/or norepinephrine.

### 5.2. Short-chain fatty acids

Short-chain fatty acids (SCFAs) are a class of low molecular weight, organic monocarboxylic acids with a carbon chain length of up to six atoms (100). Acetate (C2), propionate (C3), and butyrate (C4) are the three major SCFAs in the gut, with an approximate molar ratio of 60:20:20 (101). SCFAs are energy substrates for colonocytes and hepatocytes, except for acetate, which cannot be oxidized in the liver (102). The concentrations of SCFAs are substantially lower in the circulating system compared with the colon. It has been shown that SCFAs can cross the blood brain barrier (BBB) while maintaining their integrity, although the SCFA levels in the brain are relatively low (103, 104). Nevertheless, the SCFAs are capable of exerting systemic effect by binding to G protein-coupled receptors (GPRs) or function by playing a role as histone deacetylase (HDAC) inhibitors (105). Accumulating evidence has shown that SCFAs possess anti-inflammatory and neuroprotective capabilities to maintain a robust microglial function through the inhibition of HDACs (104, 106). Butyrate supplementation showed effects in improving long-term memory consolidation and promoting the expressions of brain-derived neurotrophic factor (BDNF) and neurogenesis in rodents (107). Exploration into the underlying mechanisms that govern the modulation of neuronal function by SCFAs has illuminated that the activation of GPR41/GPR43 may mediate some of these effects. Dalile et al. (108) examined the effects of one-week SCFA-mixture supplementation on responses to psychosocial stress in healthy adults and demonstrated that both low (87.1 mmol acetate, 6.6 mmol propionate, and 26.2 mmol butyrate) and high (174.2 mmol acetate, 13.3 mmol propionate, and 52.4 mmol butyrate) SCFA mixture significantly mitigated the cortisol-induced psychosocial stress, but did not affect subjective mood ratings (108).

In humans, depressive symptoms were positively associated with fecal acetate levels and negatively associated with both butyrate and propionate levels (109, 110). It is noteworthy that these studies investigated fecal SCFAs. Although it is a valid surrogate measure of the gut SCFAs, it may be subject to various sources of bias (104). In animals, sodium butyrate supplementation led to a reversed depressive-like behavior in animal models of depression, potentially by improving the function of the mitochondrial respiratory chain complexes and the activity of tricarboxylic acid cycle enzymes in the stratum of rats (111). Consistently, another *in vivo* study reported that sodium butyrate showed antidepressant-like activity, which was, at least partially, attributed to an increase of BDNF and inositol depletion (112, 113) and increased histone acetylation in the hippocampus (114). The effects of butyrate supplementation for preventing or alleviating depression need to be further validated in humans.

### 5.3. Inflammation

Under normal physiological circumstances, the activation of immune cells and subsequent cytokine production may elicit only modest effects within the central nervous system (CNS). Nevertheless, sustained systemic inflammation, frequently precipitated by infectious agents, has been strongly linked with a range of behavioral and cognitive impairments (115, 116). Lipopolysaccharides (LPS) are outer membrane components of gram-negative bacteria. Increased levels of circulating LPS may prompt the upregulation of pro-inflammatory cytokines, including TNFα, monocyte chemoattractant protein-1 (MCP-1), interleukin-1 beta (IL-1β), and nuclear factor kappa B p65 (NF-κB p65) (117, 118). The precise mechanism through which peripheral LPS exerts its impact on the brain remains unknown. However, one plausible route involves the translocation of LPS across the BBB. Alternatively, LPS may trigger afferent nerves beyond the BBB, operate at circumventricular organs, and induce changes in the permeability and function of the BBB (119). Interestingly, some preclinical evidence suggests that LPS is a double sword for maintaining neural homeostasis, as low-dose LPS preconditioning suppressed the production of proinflammatory cytokines, whereas a high LPS concentration may induce potent inflammatory signaling cascade (120). Peripheral insults which elicit a systemic inflammatory response may serve to promote the mobilization of peripheral immune cells toward the brain, resulting in the excessive activation of the neuroimmune response (6). Activation of Toll-like receptor 4 (TLR4) by LPS results in the secretion of inflammatory cytokines and the enhancement of costimulatory molecules on antigen-presenting cells (121). But it is important to note that although LPS is recognized for producing Th1 responses, it exhibits pleiotropic properties as it is also capable of promoting differentiation into other T helper lineages given the appropriate conditions (121).

Meta-analyses of clinical studies have indicated a strong indication of pro-inflammatory cytokine involvement in depression, as demonstrated by higher concentrations of IL-6 and TNFα in the blood of depressed patients as compared to controls (122, 123). However, another prospective study reported that inflammatory cytokines were not significantly associated with 10-year depression prognosis (124), indicating that an inflammatory status may impose acute effects on depression.

## 6. Conclusion and future directions

**Figure 3:**
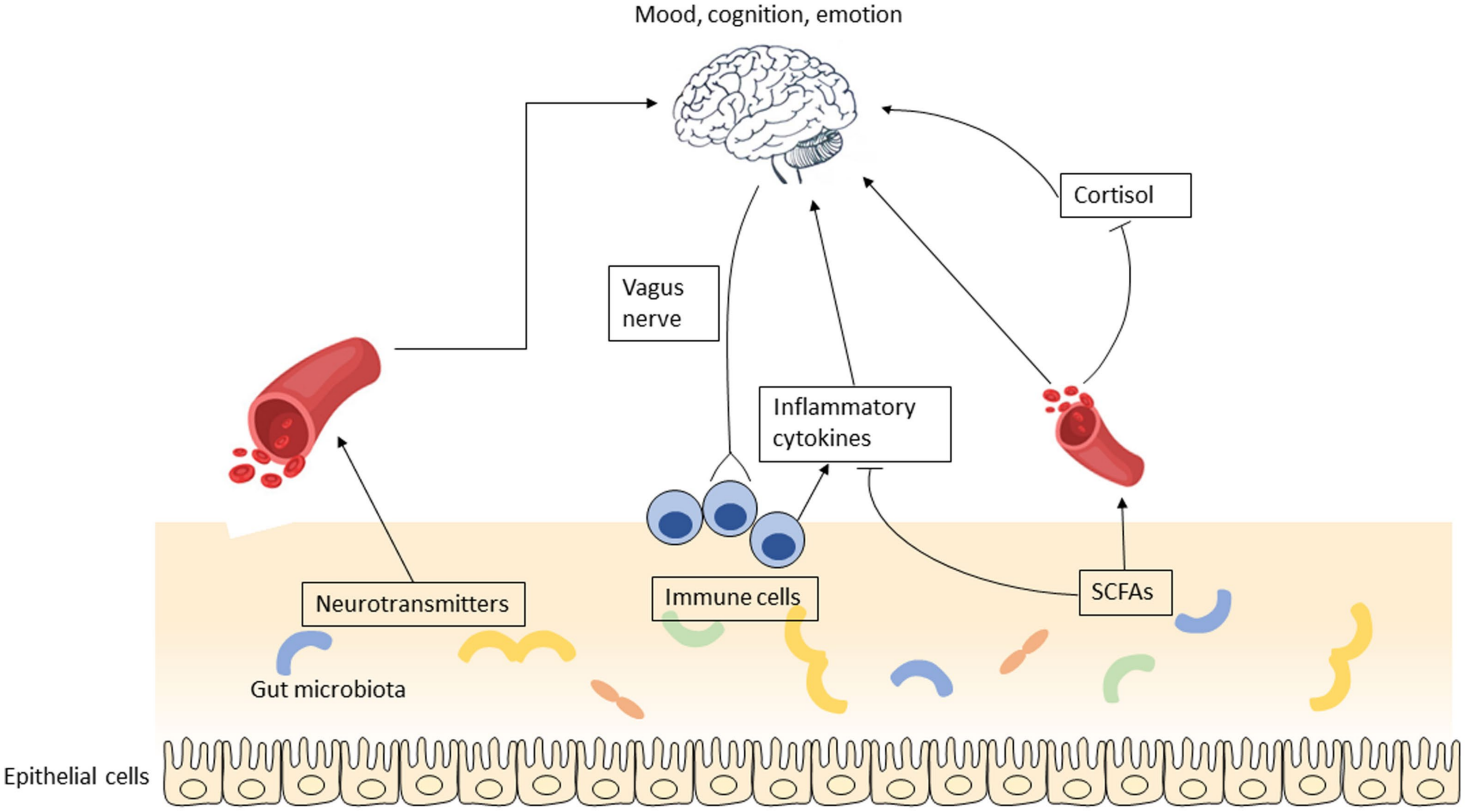
The putative mechanisms that account for the impact of prebiotics on depression involve several potential pathways, including the synthesis of neurotransmitters, production of short-chain fatty acids, and regulation of inflammation.

The current work summarized the role of promising prebiotics on modulating the development and severity of depression. By summarizing the existing clinical trials on prebiotics and depression, we concluded that GOS and n-3 PUFAs are effective in mitigating depressive symptoms. It is postulated that EGCG exhibits potential antidepressant properties, however, a higher dosage of EGCG may be required to elicit a significant effect. The plausible mechanisms that account for the impact of prebiotics are the synthesis of neurotransmitters, production of short-chain fatty acids (SCFAs), and regulation of inflammation (Figure 3). The impact of alternative prebiotics on depression has yet to be fully elucidated owing to several factors, including a dearth of clinical trials, disparate methodologies, variations in ingredient sources, and divergent extraction techniques.

While alterations in the microbial composition have been commonly documented in individuals with depression, there exists considerable variability in the specific microbiota that exhibit augmentation or reduction. The taxonomy that predominantly contributes to the onset and progression of depression is a subject of current investigation by several research teams; however, the answer to this question remains elusive. Multiple factors, such as age, gender, medication or antibiotic usage, geographical location, health status, physical activity, dietary patterns, and sleep duration, among others, may impact the diversity and profile of gut microbiota. Thus, it is crucial to record and report detailed subject characteristics to account for potential discrepancies between studies.

Considering that a beneficial microbial profile may function to prevent or alleviate depression and other mood disorders, it raises the possibility that probiotics may be functional for anti-depression as well. Prebiotics and probiotics differ in their mode of action, mechanisms, evidence, dosage, efficacy, side effects, study design, and future research directions. Prebiotics indirectly promote the growth of beneficial gut bacteria by providing nourishment, while probiotics introduce beneficial bacteria directly into the gut (125). Prebiotics affect depression through mechanisms such as neurotransmitter synthesis, short-chain fatty acid production, and inflammation regulation. Evidence for prebiotics is limited but promising, while probiotics have shown positive effects in numerous studies. Dosages vary for both, with prebiotics typically ranging from 2 to 10 g and probiotics ranging from 1 to 50 billion CFUs per day (10, 126, 127). Further research is needed to establish effectiveness, optimal dosages, and strains for both prebiotics and probiotics in depression treatment. The differences between the trials using prebiotics and postbiotics in depression are described in Table 4. A comprehensive evaluation of 10 clinical trials comprising a cumulative patient population of 1,349 subjects showed that no statistically significant difference in mood was observed between the intervention group with probiotics supplementation and the placebo group, although a subgroup analysis of studies conducted on depressed individuals exhibiting mild to moderate depressive symptoms revealed a noteworthy enhancement in the moods (127). The effects of probiotics on mitigating perinatal depression have also been controversial, and even the supplement of same probiotic strain showed different effects (6). The comparability of current clinical trials is hindered by inter-study discrepancies in probiotic dosing, bacterial strains, and strain combinations. Additionally, the generalizability of findings to depressed individuals is impeded by the fact that the majority of existing randomized controlled trials were performed on healthy populations. Nevertheless, it leads to the hypothesis that modulating the abundance of one group of gut microbiota may be insufficient to show anti-depressive effects. Thus, manipulating the gut microbiota as an integrated system by using different ingredients, such as prebiotics and fibers, may serve as a more effective approach to prevent or alleviate the development of depression.

**Table 4:** Differences between prebiotics and probiotics.

Differences	Prebiotics	Postbiotics
Definition	Non-digestible substances that stimulate the growth and activity of beneficial bacteria in the gut and confer health benefits	Live microorganisms that confer health benefits when consumed in adequate amounts
Mode of action	Indirectly promote the growth of beneficial gut bacteria through providing nourishment	Directly introduce beneficial bacteria into the gut to restore microbial balance
Mechanisms	Synthesis of neurotransmitters—Production of short-chain fatty acids—Regulation of inflammation	Modulation of gut-brain communication—Immune system modulation—Anti-inflammatory effects
Evidence	Limited number of studies exploring the effects on depression, but show promising results	More extensive research demonstrating positive effects on depressive symptoms
Dosage	Varies depending on the specific prebiotic used, typically ranging from 2 to 10 grams per day	Varied strains and dosages, commonly ranging from 1–50 billion colony-forming units (CFUs) per day
Efficacy	Mixed results, with some studies reporting improvements in depressive symptoms, while others show no effect	Overall, positive effects observed in a significant number of studies, but efficacy may vary among individuals
Side effects	Generally well-tolerated, but may cause mild gastrointestinal symptoms such as bloating or gas	Minimal side effects reported, primarily gastrointestinal discomfort in rare cases
Study design	Limited number of randomized controlled trials (RCTs)	Numerous RCTs conducted, providing stronger evidence of efficacy
Future research	More rigorous and larger-scale RCTs needed to establish effectiveness and identify optimal dosages	Further exploration of specific strains, dosages, and treatment durations to enhance efficacy and consistency

Combined prebiotic and probiotic therapy and the use of prebiotics in combination with traditional antidepressants have garnered attention as potential treatment approaches for depression (128). This synergistic approach has the potential to improve gut microbiota composition and function, which may positively impact mental health. However, a recent RCT showed that in adults experiencing moderate psychological distress and low prebiotic intake, adopting a high-prebiotic dietary intervention shows potential in enhancing mood, reducing anxiety, alleviating stress, and improving sleep. However, the combination of a high-prebiotic diet and probiotic supplement, known as a synbiotic approach, does not seem to yield beneficial effects on mental health outcomes (129). More research is needed to explore the specific combinations, dosages, and strains that would yield optimal outcomes. Additionally, the efficacy and safety of combined therapy should be thoroughly evaluated through well-designed clinical trials.

Regarding prebiotics in combination with traditional antidepressants, the rationale behind this approach lies in the potential of prebiotics to modulate gut microbiota and influence the efficacy of antidepressant medications. By improving gut health and promoting the production of beneficial metabolites, prebiotics could potentially enhance the therapeutic effects of traditional antidepressants. However, the interactions between prebiotics and specific antidepressant medications need to be further investigated to understand potential synergies or adverse effects. Additionally, individual variability and personalized treatment strategies should be considered. Both combined prebiotic/probiotic therapy and the combination of prebiotics with traditional antidepressants offer intriguing possibilities for improving treatment outcomes in depression. Nevertheless, more robust clinical trials and mechanistic studies are required to determine the optimal protocols, dosages, and mechanisms underlying these approaches. The field of psychobiotics, which explores the interaction between the gut microbiota and mental health, holds promise for advancing our understanding and refining these combination therapies for depression. Currently, there are commercially available prebiotic antidepressant preparations that aim to harness the potential benefits of prebiotics in managing depression. These products typically contain specific types of prebiotics that are believed to support a healthy gut microbiota and potentially improve mental well-being. It is important to note that while these prebiotic antidepressant preparations are available, their efficacy and safety in treating depression have not been extensively studied or established. The scientific literature on the specific formulations and their effects on mental health outcomes is limited. Individuals considering the use of commercially available prebiotic antidepressant preparations should exercise caution and consult with healthcare professionals before incorporating them into their treatment plan. It is advisable to seek evidence-based treatments and interventions that have undergone rigorous scientific evaluation and have a proven track record in managing depression.

Although the production of SCFAs is considered one of the primary mechanisms through which prebiotics alleviate depression and modulate mood, quantifying the dose of SCFAs delivered to the colon presents a significant challenge. Whole food or natural dietary sources cannot provide supraphysiological quantities of SCFAs, thereby impeding investigations into the potential benefits of SCFAs on brain function. To explore the dependent effects of SCFAs, animal studies often rely on oral administration, which may lead to distinct physiological outcomes due to the absorption of SCFAs from the upper gastrointestinal tract. Intravenous administration of SCFAs bypasses the absorption and oxidation processes that occur in colonocytes and is thus non-physiological. Additionally, rectal administration is not suitable for chronic use (103). Consequently, future studies should be undertaken to establish the optimal dosage of SCFAs delivered to the gut through prebiotic supplementation. Moreover, research into identifying prebiotic sources that produce the most abundant SCFAs would be valuable in developing dietary strategies for preventing the onset of depression.

The existing literature on the relationship between prebiotics and depression exhibits several limitations, both in terms of methodological rigor and conceptualization of future research. These limitations highlight the need for further investigation to establish a clearer understanding of this topic. Methodologically, many studies suffer from small sample sizes, limiting the generalizability of their findings. Depression is a complex and multifaceted condition, and studying it requires large and diverse participant groups to capture its full spectrum. Moreover, the majority of studies rely on self-report measures for assessing depression symptoms, which can introduce biases and inaccuracies. The use of more objective and standardized diagnostic criteria, such as structured clinical interviews, would enhance the reliability of the results. Another limitation lies in the inconsistency of prebiotic interventions across studies. There is a lack of consensus on the optimal dose, duration, and type of prebiotics to administer. This heterogeneity hampers the ability to compare findings across studies and draw definitive conclusions. Future research should strive for standardized protocols to facilitate more meaningful comparisons and meta-analyses. Additionally, most studies focus solely on the effects of prebiotics on depressive symptoms, neglecting potential underlying mechanisms. Exploring the gut-brain axis and investigating the specific pathways through which prebiotics influence brain function would provide a more comprehensive understanding of their antidepressant effects. Last but not least, the generalizability of the findings might be impeded because the majority of existing studies were conducted on diseased population, so it would be challenging to leverage the current findings to indicate how healthy individuals may benefit from consuming the prebiotics. Overall, the existing literature on prebiotics and depression is limited by methodological shortcomings, including small sample sizes and inconsistencies in intervention protocols. Moving forward, it is essential to address these limitations by employing larger, more diverse samples, utilizing longitudinal designs, establishing standardized intervention protocols, and investigating underlying mechanisms. By addressing these gaps, future research can advance our knowledge of the potential role of prebiotics in the treatment and prevention of depression.

In conclusion, the results of our study indicate that a daily intake of 5 g of GOS and 1 g or less of EPA can be effective in alleviating depressive symptoms. Although EGCG shows promise as an antidepressant, a higher dosage of 3 g per day might be required to achieve notable effects. The potential mechanisms that explain the influence of prebiotics on depression include the synthesis of neurotransmitters, the production of short-chain fatty acids, and the regulation of inflammation.

## Author contributions

YY and BZ performed literature search and data collection. YY, BZ, SZ, LS, FL, and XL conducted the study design and wrote the manuscript. FL and XL provided scientific proofreading and supervised the study. All authors contributed to the article and approved the submitted version.

## Conflict of interest

The authors declare that the research was conducted in the absence of any commercial or financial relationships that could be construed as a potential conflict of interest.

## Publisher’s note

All claims expressed in this article are solely those of the authors and do not necessarily represent those of their affiliated organizations, or those of the publisher, the editors and the reviewers. Any product that may be evaluated in this article, or claim that may be made by its manufacturer, is not guaranteed or endorsed by the publisher.
